# Refractory Alopecia Areata of the Beard: Novel Improvement Through Exosome Therapy With Signs of Hair Repigmentation

**DOI:** 10.1002/ccr3.71558

**Published:** 2025-12-05

**Authors:** Asem Shadid, Khalid Nabil Nagshabandi, Danah K. AlRuhaimi, Abdullah Al‐Omair, Abdulaziz Alsadhan

**Affiliations:** ^1^ Department of Dermatology King Fahad Medical City Riyadh Saudi Arabia; ^2^ Department of Dermatology, College of Medicine King Saud University Riyadh Saudi Arabia; ^3^ Department of Dermatology Prince Sultan Military Medical City Riyadh Saudi Arabia; ^4^ Department of Dermatology Security Forces Hospital Riyadh Saudi Arabia

**Keywords:** alopecia areata, exosomes, hair loss, regenerative medicine, stem cells

## Abstract

Exosome injections derived from human umbilical cord, with or without polydeoxyribonucleotide, induced robust regrowth, repigmentation of previously white beard hairs, and scar improvement in a 35‐year‐old man with refractory alopecia areata of the beard, suggesting a promising minimally invasive regenerative option.

## 1 Introduction

Alopecia, or baldness, is frequently diagnosed in clinical settings. It can manifest as either scarring or non‐scarring types. Alopecia areata (AA), an autoimmune disorder characterized by patchy non‐scarring alopecia, can cause hair loss in focal areas, the entire scalp, beard, eyelashes and brows, or the entire body [1]. It continues to challenge the boundaries of dermatological treatment. Traditional therapies, such as intralesional corticosteroids and immunotherapy, often yield variable results and may not be suitable for all patients, particularly those with extensive or refractory cases [1].

In recent years, the emerging field of regenerative medicine has introduced novel approaches to treat conditions like androgenetic alopecia and alopecia areata, with exosome therapy showing promising potential [2, 3]. Exosomes, which are extracellular vesicles produced from stem cells, have been found to be key mediators of cell communication. They carry proteins, lipids, and nucleic acids that can influence inflammatory responses, promote tissue regeneration, and modify immunological signaling [4]. These vesicles have been demonstrated to promote hair growth by increasing cellular proliferation and differentiation, decreasing apoptosis in follicular cells, and regulating the local immunological environment, which is frequently disturbed in alopecia areata [3]. Their significance in hair regeneration has gained attention due to their capacity to impact hair follicle behavior without the complications and ethical considerations involved with direct stem cell therapy [4]. Herein, we report a novel case of refractory AA of the beard exhibiting promising results after injecting exosomes, along with observing signs of hair repigmentation.

## 2 Case History/Examination

**FIGURE 1:**
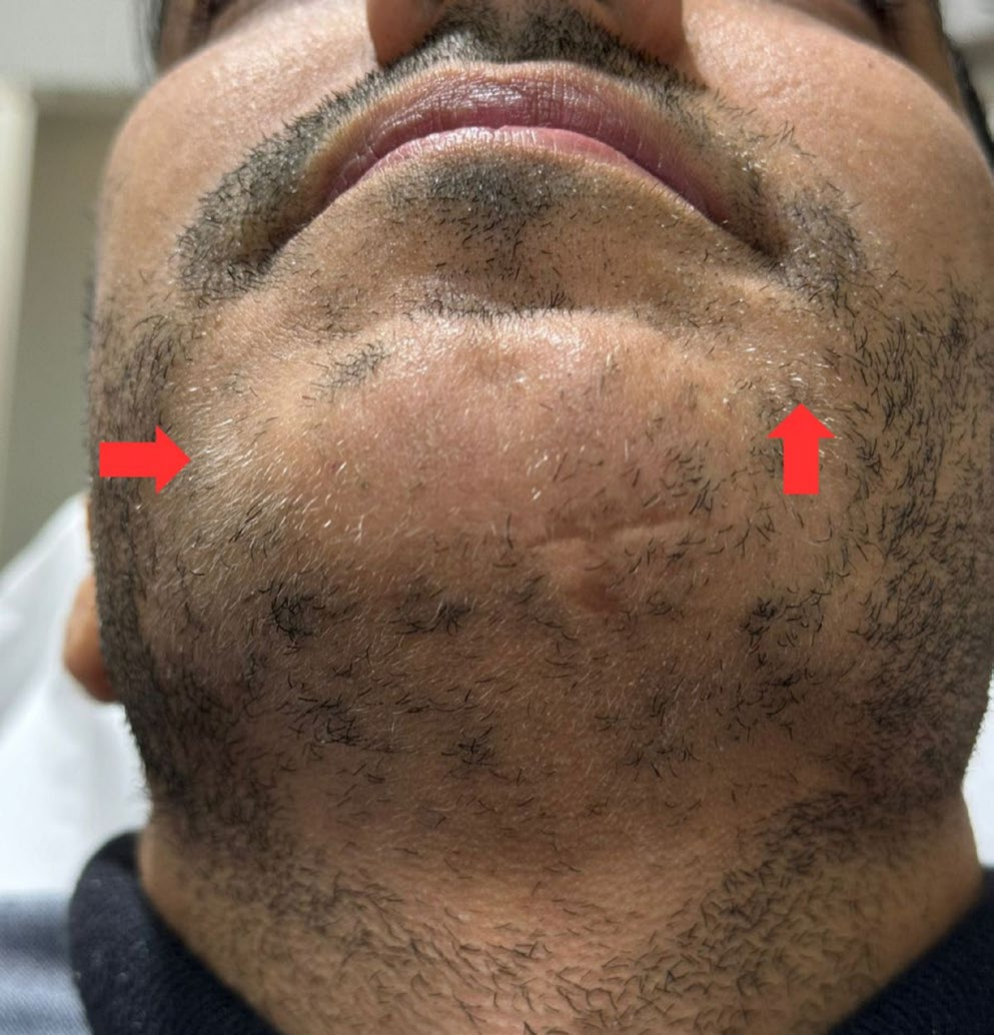
Multiple, well‐demarcated alopecic patches across the whole beard area, with an evident scar on the left lower area of the chin (Red arrow). White grayish hairs (poliosis).

A 35‐year‐old male patient with a 4‐year history of alopecia areata (AA) affecting his beard presented at our clinic. The patient had previously undergone multiple treatments, including intralesional corticosteroids (2.5 and 5 mg/mL) and topical minoxidil 5%, without achieving satisfactory results. At the time of presentation, the patient had discontinued these treatments for more than a year. On physical examination, several well‐defined alopecic patches were observed across his beard, with no signs of inflammation, erythema, or scarring. The patient also had areas of poliosis (white hairs) in the beard and a noticeable scar on the lower lip chin area (Figure 1).

## 3 Methods

**FIGURE 2:**
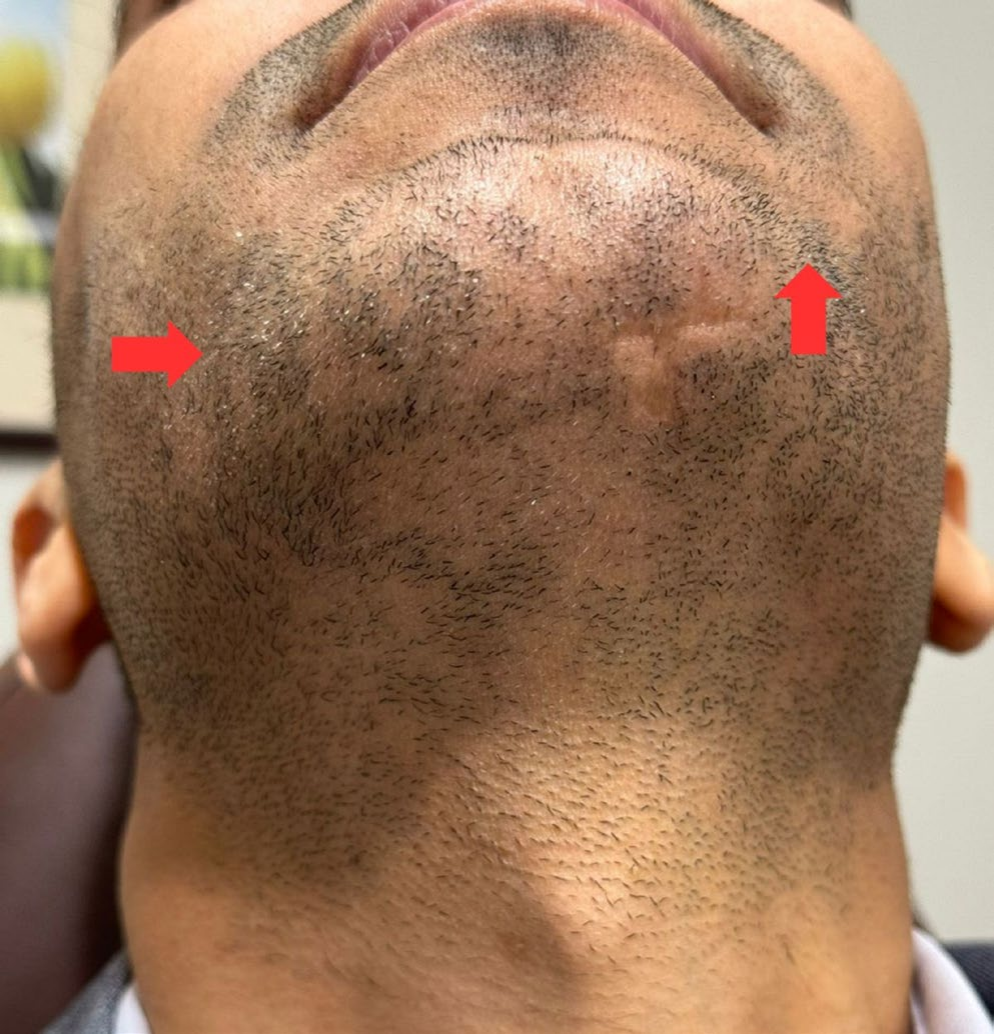
6‐week post‐injection of human umbilical cord‐derived exosomes (ASCE+ HRLV), the treated regions showed beard hair regrowth and early signs of hair repigmentation (Red arrow).

The patient's diagnosis of refractory alopecia areata had been established based on clinical findings and treatment resistance. Differential diagnoses such as tinea barbae, and cicatricial alopecia were ruled out based on the absence of inflammation, and scarring beyond the poliosis. Given the failure of conventional treatments and the patient's preference for alternative options, exosome therapy was proposed. In the first session, the patient received 3 mL/cc stem cell derived human‐based exosomes (ASCE+ HRLV exosomes for hair restoration), injected into the alopecic patches. Six weeks later, early signs of hair regrowth and repigmentation were noted (Figure 2). Based on this favorable response, and with the aim of potentially enhancing outcomes, a second session was conducted using an equivalent 3 mL of human‐based umbilical cord‐derived exosomes combined with polydeoxyribonucleotide (PDRN) (DERMAFIRM Rx Elysee Exosome Ampoule Skin Booster). The inclusion of PDRN was chosen due to its known regenerative and anti‐inflammatory properties, hypothesized to synergize with the exosomes and further stimulate follicular regeneration and scar improvement.

## 4 Results

**FIGURE 3:**
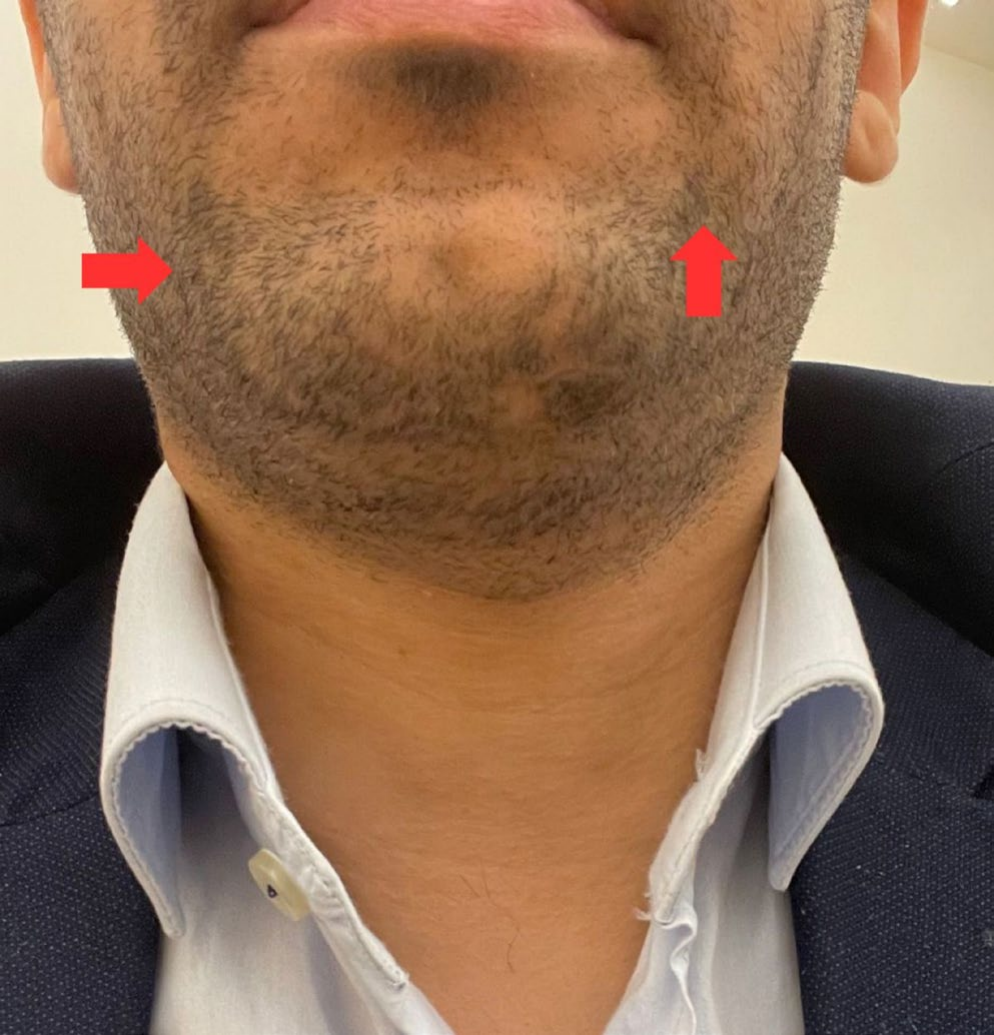
After only two injections of exosomes, significant complete hair regrowth of affected regions, along with signs of complete hair repigmentation (Red arrow). Significant improvement of the scar appearance located on the left lower chin.

Following two sessions of exosome therapy, the patient exhibited significant hair regrowth and the repigmentation of white hairs (poliosis) into black. Additionally, marked improvement in the appearance of a scar in the chin area was observed (Figure 3). Three months post‐treatment, the results were maintained, with no signs of relapse. The patient was advised to return for additional treatments as needed. This outcome suggests that exosome therapy offers a promising and minimally invasive treatment option for refractory alopecia areata, especially in cases unresponsive to conventional therapies.

## 5 Discussion

Alopecia areata (AA) is an autoimmune disorder characterized by non‐scarring hair loss that can occur anywhere on the body. It affects around 2% of the population at some point in their lives. AA is generally caused by a breakdown in the hair follicle's immunological privilege, which results in lymphocytic infiltration surrounding the hair follicle's bulb [1, 3]. Despite the availability of several therapeutic options, including corticosteroids (topical, oral, and intralesional), minoxidil, tacrolimus, contact immunotherapies (such as squaric acid dibutyl ester and diphencyprone), and photochemotherapy with UVA and psoralens, there is currently no cure for AA [5]. These treatments frequently provide unpredictable results, do not provide a lasting remedy, and can be associated with serious side effects [3, 5].

Exosome therapy has emerged as a promising treatment for alopecia areata and androgenetic alopecia (AGA), taking advantage of the regenerative properties of these extracellular vesicles. Research indicates that exosomes derived from mesenchymal stem cells (MSCs), including those obtained from umbilical cord sources, contain bioactive molecules like microRNAs, proteins, and lipids that activate key signaling pathways critical for hair regeneration, such as the Wnt/β‐catenin pathway. This pathway is crucial in regulating hair follicle development and maintaining the hair cycle, particularly in transitioning from the resting (telogen) to the active (anagen) phase [6, 7]. Umbilical cord‐derived exosomes offer an advantage by enhancing these molecular mechanisms without the challenges associated with direct cell transplantation, such as rejection or ethical concerns [6]. Adipose‐derived exosomes, which are separated from fat cells, contain growth factors, cytokines, and genetic material that can influence cellular communication and promote tissue regeneration. These exosomes have been found to be effective in increasing hair follicle proliferation, reducing apoptosis, and creating a favorable local immune environment, thereby addressing the autoimmune aspects of AA and the follicular miniaturization seen in androgenetic alopecia [3, 8]. Furthermore, plant‐derived exosomes have shown significant potential in promoting skin and hair health, as evidenced by recent studies [9]. In particular, ginseng and green tea‐derived exosomes have shown promising results in enhancing hair follicle development and promoting hair growth. These exosomes were found to increase the proliferation and differentiation of hair follicle cells while reducing cell death, thereby supporting healthier and more robust hair growth. Additionally, their anti‐inflammatory and antioxidant properties further contribute to improving scalp health and reducing hair loss [10].

A recent study by Lueangarun et al. explored the therapeutic potential of exosomes combined with fractional picosecond laser treatment in a patient with androgenetic alopecia who also experienced poliosis circumscripta, a condition characterized by localized hair depigmentation (white or gray hair). The patient showed significant hair repigmentation in the areas affected by poliosis circumscripta after the combined treatment [11]. This was also evident in our highlighted case that resulted in the repigmentation of beard hairs after only two sessions of exosome monotherapy. This outcome suggests that exosome therapy can effectively stimulate melanocyte activity in hair follicles, thereby restoring hair color along with improving hair density and health. In addition, exosome therapy also showed significant outcomes in wound healing and reducing scarring. A recent review discussing the novel mechanisms and applications of exosomes in dermatology and cutaneous aesthetics found that exosomes derived from mesenchymal stem cells have shown remarkable potential in wound healing. They promote the proliferation and migration of dermal fibroblasts and keratinocytes, enhance collagen synthesis, and facilitate angiogenesis, thereby accelerating the healing process [12]. Linking this to our case, exosomes have demonstrated their potential in both promoting hair regrowth and improving scarring, underscoring their versatility and efficacy in dermatological treatments.

Polydeoxyribonucleotide (PDRN) has gained attention in regenerative medicine, including hair restoration, due to its ability to promote tissue repair and cell regeneration. PDRN is a compound extracted from the DNA of salmon sperm, and its mechanism of action revolves around stimulating tissue repair, enhancing angiogenesis, and reducing inflammation, all of which can have direct or indirect benefits on hair growth [13]. In our case, the second course injection included exosomes that were combined with PDRN; the synergistic effects of these two agents likely played a pivotal role in the enhanced hair regrowth and in enhancing scar appearance observed [13, 14, 15]. PDRN's regenerative capabilities, particularly its ability to promote angiogenesis by activating A2A adenosine receptors, were complemented by the exosomes' ability to stimulate cellular repair and reduce follicular apoptosis. PDRN's anti‐inflammatory properties would have further supported the exosomes' function by reducing immune‐mediated damage in the alopecic areas, a key issue in refractory alopecia areata. This combination not only promoted hair density but also facilitated the repigmentation of previously depigmented hairs (poliosis), as PDRN enhanced vascularization while exosomes stimulated melanocyte activity within the hair follicles. This notable improvement highlights the effectiveness of integrating PDRN with exosome therapy, creating a regenerative microenvironment that supports follicular growth.

The application of exosome therapy in dermatology is gaining popularity due to its minimally invasive nature and the ability to harness regenerative properties without introducing live cells into the patient, thus minimizing the risk of rejection and other complications associated with cell‐based therapies. This case illustrates the potential of exosome therapy as an effective treatment modality for cases of alopecia areata that are resistant to conventional treatments. The application of human‐based umbilical cord‐derived exosomes has shown promising results in promoting hair regrowth along with signs of hair repigmentation. As research continues to evolve, the integration of exosome therapy into the treatment landscape for alopecia areata represents a promising frontier that could redefine therapeutic strategies for this unpredictable and often distressing condition. This not only offers hope for patients with refractory AA but also underscores the potential of regenerative medicine in addressing autoimmune and inflammatory conditions more broadly. Moreover, larger sample size studies are required to corroborate these initial positive findings and further understand the mechanisms underlying the observed hair repigmentation.

## 6 Conclusion

Exosome therapy has shown promising results in a patient with refractory alopecia areata of the beard. The observed hair regrowth, repigmentation, and scar improvement after exosome injections highlight the potential of this innovative treatment. As regenerative medicine continues to evolve, exosome therapy may emerge as a valuable option for patients with recalcitrant alopecia areata. Further studies with larger sample sizes are warranted to explore the long‐term efficacy and safety of exosome therapy in dermatological conditions such as alopecia areata.

## Author Contributions

**Asem Shadid:** conceptualization, data curation, formal analysis, funding acquisition, investigation, methodology, project administration, visualization, writing – original draft, writing – review and editing. **Khalid Nabil Nagshabandi:** conceptualization, data curation, formal analysis, funding acquisition, investigation, methodology, project administration, resources, visualization, writing – original draft, writing – review and editing. **Danah K. AlRuhaimi:** conceptualization, data curation, formal analysis, funding acquisition, investigation, methodology, project administration, visualization, writing – original draft, writing – review and editing. **Abdullah Al‐Omair:** conceptualization, data curation, formal analysis, funding acquisition, investigation, methodology, project administration, visualization, writing – original draft, writing – review and editing. **Abdulaziz Alsadhan:** conceptualization, data curation, formal analysis, funding acquisition, investigation, methodology, project administration, resources, supervision, visualization, writing – original draft, writing – review and editing.

## Funding

The authors have nothing to report.

## Ethics Statement

The authors have nothing to report.

## Consent

Written informed consent was obtained from the patient for publication of the details of their medical case and any accompanying images.

## Conflicts of Interest

The authors declare no conflicts of interest.

## Data Availability

All data generated or analyzed during this study is included in this article. Further enquiries can be directed to the corresponding author.
