# Correction to “Refractory Alopecia Areata of the Beard: Novel Improvement Through Exosome Therapy With Signs of Hair Repigmentation”

**DOI:** 10.1002/ccr3.71703

**Published:** 2025-12-21

**Correction to:**

Shadid A, Nagshabandi KN, AlRuhaimi DK, Al‐Omair A, Alsadhan A. Refractory Alopecia Areata of the Beard: Novel Improvement Through Exosome Therapy With Signs of Hair Repigmentation. *Clinical Case Reports*. 2025;13:e71558. 10.1002/ccr3.71558.

we would like to request the following corrections:

Abstract

**Published text (first sentence)**

The abstract currently describes our treatment as follows:

“Exosome injections derived from human umbilical cord, with or without polydeoxyribonucleotide.”

**Corrected text**

This should be read as:

“Exosome injections consisting of human adipose‐derived mesenchymal stem cell exosomes (ASCE+ HRLV) and plant‐derived exosomes (DERMAFIRM Rx Elysee Exosome Ampoule Skin Booster)”.

The remainder of the sentence in the abstract should remain unchanged.

Methods—ASCE+ HRLV Exosomes (First Treatment Session).

**Published text**

In the Methods section, ASCE+ HRLV in the first session are described as:

“stem cell derived human‐based exosomes”.

**Corrected text**

This should be read as:

“human adipose‐derived mesenchymal stem cell exosomes (ASCE+ HRLV exosomes for hair restoration)”.

This correction clarifies that ASCE+ HRLV are not umbilical cord–derived, but rather human adipose‐derived mesenchymal stem cell exosomes used for scalp/hair restoration.

Methods—DERMAFIRM Rx Elysee Exosome Ampoule Skin Booster (Second Treatment Session).

**Published text**

In the Methods section, the second treatment session is described as using:

“human‐based umbilical cord‐derived exosomes combined with polydeoxyribonucleotide (PDRN) (DERMAFIRM Rx Elysee Exosome Ampoule Skin Booster)”.

**Corrected text**

This should be read as:

“plant‐derived exosomes (DERMAFIRM Rx Elysee Exosome Ampoule Skin Booster)”.

We also wish to clarify that while other products in the Elysee line from the same manufacturer may be based on umbilical cord–derived exosomes, the product used in our case report in Saudi Arabia was the **plant‐derived exosome** ampoule as specified above.

These corrections concern only the classification and description of the exosome products. They do not alter the clinical course, results, or conclusions of our report. We kindly request that an official correction/erratum be issued to reflect these points, and we would be grateful if the journal could confirm the commercial names and sources of the products as part of the editorial correction process.

Thank you very much for your assistance. We apologize for this error.

